# Heavy Traffic Feasible Hybrid Intracycle and Cyclic Sleep for Power Saving in 10G-EPON

**DOI:** 10.1155/2014/497379

**Published:** 2014-08-11

**Authors:** Xintian Hu, Liqian Wang, Zhiguo Zhang, Xue Chen

**Affiliations:** State Key Lab of Information Photonics and Optical Communications, Beijing University of Posts and Telecommunications, Beijing 100876, China

## Abstract

Energy consumption in optical access networks costs carriers substantial operational expense (OPEX) every year and is one of contributing factors for the global warming. To reduce energy consumption in the 10-gigabit Ethernet passive optical network (10G-EPON), a hybrid intracycle and cyclic sleep mechanism is proposed in this paper. Under heavy traffic load, optical network units (ONUs) can utilize short idle slots within each scheduling cycle to enter intracycle sleep without postponing data transmission. In this way, energy conservation is achieved even under heavy traffic load with quality of service (QoS) guarantee. Under light traffic load, ONUs perform long cyclic sleep for several scheduling cycles. The adoption of cyclic sleep instead of intracycle sleep under light traffic load can reduce unnecessary frequent transitions between sleep and full active work caused by using intracycle sleep. Further, the Markov chain of the proposed mechanism is established. The performances of the proposed mechanism and existing approaches are analyzed quantitatively based on the chain. For the proposed mechanism, power saving ability with QoS guarantee even under heavy traffic and better power saving performance than existing approaches are verified by the quantitative analysis. Moreover, simulations validate the above conclusions based on the chain.

## 1. Introduction

Due to the global warming, there are increasing interests in reducing energy consumption in many fields including telecommunication networks [1, 2]. The information and communication technology (ICT) took up 8% of worldwide electricity in 2009 [3]. This value is still growing rapidly as the speed of communication increases. Access networks are the last mile between users and core networks. As an optical access architecture, it is generally considered that passive optical network (PON) is of low energy consumption, owing to the usage of passive components along with fibers [4]. However, the 15% energy utilization of PON is much lower than that of metro and core networks [5]. PON provides services to thousands of subscribers. The huge number of nodes also results in tremendous energy consumption [6, 7]. Power saving in PON is of huge potential and can reduce operational expenditure (OPEX) for carriers.

**Figure 1:**
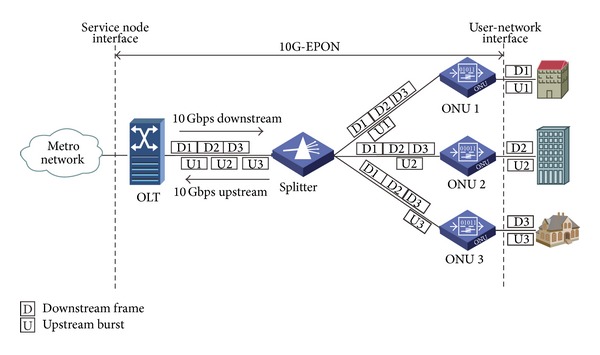
Downstream and upstream data transmission in 10G-EPON.

PON has become the most promising technology of access networks and achieved large-scale deployment worldwide [8–10].  Figure 1 shows the data transmission manner in the time division multiplexing (TDM) PON, such as 10-gigabit Ethernet passive optical network (10G-EPON). 10G-EPON is composed of one optical line terminal (OLT) and several optical network units (ONUs). Passive optical fibers and optical splitters connect the OLT and ONUs. The downstream transmission and upstream transmission are based on TDM and time division multiple access (TDMA). In the downstream direction, the OLT broadcasts data to all ONUs. Each ONU identifies its own data based on logic link identification (LLID). In the upstream direction, the dynamic bandwidth allocation (DBA) is performed in the OLT to allocate bandwidth resource to ONUs effectively and avoid transmission collision. By the request and guarantee protocol, ONUs obtain allocation results of the DBA and send data to the OLT in nonoverlapping transmission windows.

The problem of TDM PON is that ONUs always keep in active state to receive broadcasted traffic from the OLT and discard received data of other ONUs [13]. A great amount of energy is wasted in receiving that discarded traffic. Therefore, a direct way for power saving is to make ONUs shut down active elements and enter sleep mode of low energy consumption when ONUs are not the destination of any traffic.

In recent years, many studies of power saving have been proposed based on sleep mechanism [14, 15]. In G.987.3, the ITU-T mentions the cyclic sleep mechanism [16]. ONUs enter sleep mode of low power consumption under light traffic and periodically wake up. When ONUs wake up, the OLT helps ONUs to check whether it should remain asleep [17, 18]. Further, to guarantee the quality of service (QoS) of high priority applications, a prequitting method is proposed to cooperate with sleep mechanism [11, 19]. In the scheme, ONUs themselves can quit sleep mode before the end of preset sleep duration. When high priority traffic arrives, ONUs prequit sleep and receive the GATE frames from the OLT. In the GATE frames, the OLT reserves upstream bandwidth for ONUs in sleep mode and reserved bandwidth can at least hold a REPORT message. ONUs use the reserved bandwidth to report their bandwidth request and recover transmission in time. In the scenes of large downstream traffic and little upstream traffic, ONUs can perform dozing [20]. In dozing mode, ONUs only power off transmitters and keep receivers active. More opportunities for reducing energy consumption are created. ITU-T G.Sup45 [20] introduces deep sleep and power shedding too. The principle of sleep by powering off devices or elements during idle durations is also applied to other devices, such as Ethernet aggregator (EA) [21].

Besides sleep-based schemes, Kubo et al. proposed an adaptive link rate (ALR) mechanism to complement cyclic sleep [22]. Cyclic sleep is effective against bursty traffic, but the ALR is used to cope with smooth traffic. According to the actual traffic load, the ALR controls the PON system to switch between 1 Gbps and 10 Gbps. Therefore, based on the belief that low-rate link consumes less power than high-rate link, power saving can be achieved when line rate switches into 1 Gbps. Alcatel-Lucent reported a scheme called bit interleaving passive optical network (BIPON) [23]. In BIPON, power saving is achieved by adjusting protocol. The downstream frame structure is modified. Bits of different ONUs in the frame are organized in a bit-interleaved pattern. In this way, one ONU can get its data by extracting bits periodically and does not need to resolve all bits. Thus, the majority of function modules in ONUs do not need to operate at full line rate any more. Experiments prove that BIPON can reduce the energy consumption of ONUs to one-thirtieth of the original value.

Moreover, in next generation PON, the attention of power saving may transfer to optimization of network architecture and utilize agile wavelength division multiplexing (WDM) technology [24, 25].

In this paper, attentions are focused on solving the problem of the popular sleep-based power saving methods. These methods tend to perform long sleep and are only effective under light traffic. Under heavy traffic load, if ONUs enter sleep mode for a long time, fast arriving packets will quickly fill in the caches. This leads to the increase of traffic delay and loss of packets. The QoS cannot meet users' requirement.

To solve the above problems, a hybrid intracycle and cyclic sleep mechanism is proposed. Under heavy traffic load, ONUs, which can quickly wake up from sleep, perform intracycle sleep by utilizing short idle slots among sending and receiving windows. In this way, even under heavy load ONUs can perform short sleep without postponing data transmission. However, to perform intracycle sleep, ONUs need to interact with the OLT and have to frequently switch between full active work and sleep. Therefore, under light load, ONUs perform long cyclic sleep. This is to save energy by reducing unnecessary interactions between the OLT and ONUs. In conclusion, the hybrid sleep can reduce energy consumption even under heavy load and improve the power saving performance under light load. In addition, to guarantee the delay of high priority applications ONUs can prequit sleep [11, 19].

The rest of this paper is organized as follows. The detailed motivations and design of the hybrid sleep mechanism are discussed in Section 2. In Section 3, mathematical model of energy consumption with hybrid sleep is established. The performance of the hybrid sleep is analyzed and further evaluated by comparison with existing cyclic sleep mechanism and pure intracycle sleep mechanism. In Section 4, via simulations the accuracy of the mathematical model is validated and the performance of the hybrid sleep with prequitting method is shown. Finally, conclusions are drawn in Section 5.

## 2. Proposed Hybrid Intracycle and Cyclic Sleep Mechanism

In this section, the motivations, basic operations, ONU states, and algorithms run in OLT of the proposed hybrid sleep mechanism are discussed in detail.

### 2.1. Motivations and Basic Operations of the Proposed Mechanism

The main idea of the hybrid sleep mechanism is to combine the advantages of both intracycle sleep and cyclic sleep.

#### 2.1.1. Intracycle Sleep

**Figure 2:**
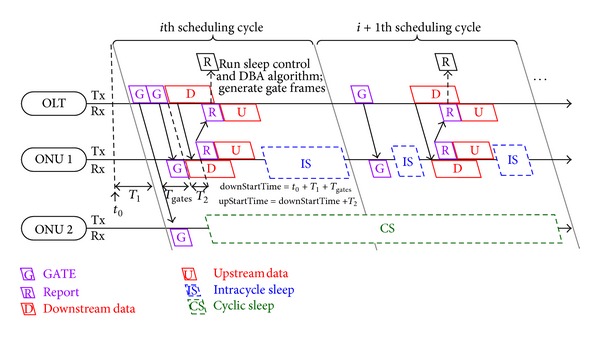
Interaction and operations of the OLT and ONUs.

For intracycle sleep, ONUs utilize short idle durations that are durations before or after upstream and downstream data transmission to perform sleep. The idle durations, quadrangular areas surrounded by dashed line and labeled by IS (intracycle sleep) in Figure 2, exist even in the busiest cycles. Actually, in TDM PON, at most two ONUs can perform data transmission simultaneously. One ONU is sending data while the other ONU is receiving data. Except for the two ONUs, all other ONUs are idle. Therefore, even under heavy load ONUs can perform short sleep in the idle pieces while they still hold their transmission windows in each scheduling cycle.

Based on fast clock and data recovery (CDR) circuit, ONUs have the ability to perform sleep in the short idle pieces and wake up in time. According to IEEE 802.3 av, the wake-up time of ONUs from sleep to full active work includes laser on time, receiver setting time, clock recovery time, and frame synchronization time. The laser on time and receiver setting time are less than 1 *μ*s. The frame synchronization time can be ignored by making ONUs wake up just before transmission windows. Based on fast CDR circuit, the clock recovery time is less than 500 ns [26]. Therefore, the total wake-up time (*T*_wakeup_), obtained from Table 1, is limited to 2 *μ*s and this enables short intracycle sleep within ms-level scheduling cycles. Note that the fast CDR circuits use similar manufacturing process and materials as original ones. Compared to optical transceivers, CDR circuits are not the main cost parts of ONUs. The fast CDR circuits do not increase the cost of ONUs significantly.

#### 2.1.2. The Mixture of Intracycle Sleep and Cyclic Sleep

The weakness of intracycle sleep is that ONUs have to wake up and keep in full active state at the beginning of each cycle to receive instructions from the OLT. Moreover, after the reception of instructions ONUs can perform intracycle sleep, but ONUs may wake up again to send and receive data during each cycle. The energy is wasted in frequently wake-up and fall-asleep process, especially when ONUs can perform long sleep over several cycles under no or little traffic. Therefore, to improve the power saving performance, long cyclic sleep should be assigned to ONUs under no or little traffic.

#### 2.1.3. Basic Operations

Figure 2 shows the information exchanges and operations of the OLT and ONUs in the hybrid sleep mechanism. The hybrid sleep mechanism is proposed in 10G-EPON. Symmetrical 10 Gbps upstream and downstream line rates are selected. In the proposed mechanism, the OLT acts as the master and controls the behaviors of all ONUs with fixed scheduling cycle (*T*_cycle⁡_). Also, the OLT arranges sleep and data transmission for all ONUs together to achieve better power saving performance. Since the bandwidth scheduling scheme is not specified in IEEE 802.3 av, the above settings are allowed.

The OLT broadcasts the GATE frames and downstream data to all ONUs. Both sleep control information and upstream transmission arrangement are contained in the GATE frames. There are no ratified bits for carrying sleep control information, so reserved bits in the GATE frames are used [27]. Except for ONUs in long cyclic sleep, ONUs keep receiving the GATE frames for designated time (*T*_gates_) at the beginning of each scheduling cycle. *T*_gates_ should be long enough for the farthest ONU to receive the last GATE frame. According to indications in the GATE frames, ONUs may send bandwidth request REPORTs and upstream data, receive downstream data, perform intracycle sleep, or execute cyclic sleep. The OLT collects all upstream REPORTs from ONUs and receives upstream data. Then, the OLT runs the sleep control and DBA algorithm to determine operations of the next scheduling cycle for ONUs. The GATE frames are generated after running the algorithm. The downstream data transmission is also arranged by the sleep control and DBA algorithm.

### 2.2. States and Power Consumption of ONUs

**Figure 3:**
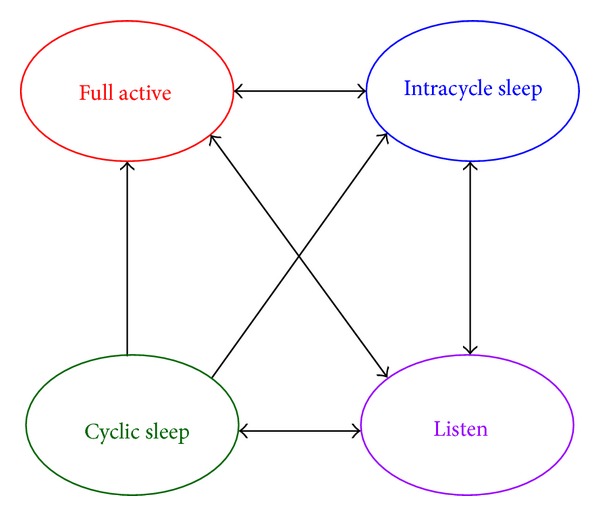
State transitions of an ONU.

The state transitions are shown in Figure 3. Each ONU switches among full active work state, intracycle sleep state, listening state, and cyclic sleep state. The OLT controls the state transitions. The state transitions are based on two thresholds. The intracycle sleep threshold (*M*) is used to control the entrance and exit of intracycle sleep, while the cyclic sleep threshold (*N*) is used to determine the entrance and exit of cyclic sleep. To perform intracycle sleep under heavy traffic and cyclic sleep under light traffic, intracycle sleep threshold should be larger than cyclic sleep threshold. Full active work state is assigned to an ONU when the bytes stored in the downstream cache or recorded in the bandwidth request REPORT are larger than intracycle sleep threshold. When both downstream and upstream bandwidth demands are less than cyclic sleep threshold, listening state is first allocated to an ONU. Then, an ONU can enter cyclic sleep state from listening state when demands are still less than cyclic sleep threshold. An ONU in cyclic sleep state may be assigned to the other three states after the end of *K* continuous sleeping cycles. When the above conditions to enter full active work, listening, and cyclic sleep state are not met, intracycle sleep state is assigned to an ONU.

#### 2.2.1. Full Active Work State

In the full active work state, ONUs wait for the GATE frames from the OLT at the beginning of each scheduling cycle. The GATE frames inform ONUs of the starting time and sizes of downstream and upstream transmission windows. In assigned windows, ONUs send prepared upstream traffic and receive destined downstream data. In the process, upstream data of different priorities are stored in logically independent queues. ONUs send upstream data after bandwidth requests. Data of higher priorities are sent first. During the whole state, ONUs do not perform sleep.

#### 2.2.2. Intracycle Sleep State

In the intracycle sleep state, besides information about transmission windows, ONUs learn how to perform intracycle sleep from the GATE frames. In the proposed mechanism, ONUs adopt intracycle sleep in two types of idle durations. The first one appears after all ONUs receive the GATE frames, and the second one appears between the end of both upstream and downstream data transmission and the beginning of next cycle as shown in Figure 2. However, when the available length of one idle duration is less than the sum of wake-up time and fall-asleep time (*T*_fallasleep_), intracycle sleep during the idle duration is not assigned to the ONU. For example, in the (*i* + 1)th cycle in Figure 2, ONU 1 performs intracycle sleep in both idle durations, but in the *i*th cycle, ONU 1 only performs intracycle sleep in the second idle duration. To enter intracycle sleep, ONUs turn off transceivers, keeping timing function and cache data from user-network interfaces (UNIs). To perform data transmission after sleep, ONUs wake up and recover synchronization with the OLT before the start of transmission windows.

#### 2.2.3. Listening State

ONUs must experience listening state to enter cyclic sleep state. Without listening state, ONUs may enter long sleep under heavy load when the arrival rate falls to low value for an instant. After the low-rate instant, arrival rate returns to high value, but ONUs fall asleep. Fast arriving packets will fill in caches and QoS goes bad. The duration of listening state is set to one cycle in this paper. Except for controlling the entrance of cyclic sleep state, the operations in listening state are similar as intracycle sleep state.

#### 2.2.4. Cyclic Sleep State

In the cyclic sleep state, ONUs fall asleep based on the GATE frames from the OLT. After sleeping for *K* cycles, ONUs wake up before the start of the (*K* + 1)th cycle. Based on the configurations, ONUs can prequit sleep by themselves. When upstream high priority traffic arrives, ONUs quit sleep and keep in active state until the start of next cycle. Then, in the next cycle ONUs make state transitions based on the GATE frames and use reserved bandwidth in the GATE frames to recover transmission.

#### 2.2.5. Power Consumption of Different States

**Figure 4:**
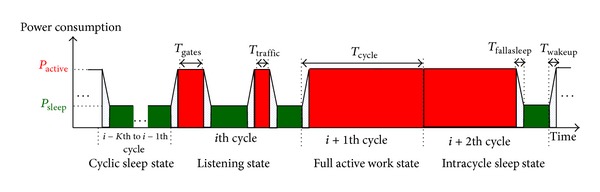
Possible power transitions of an ONU in different states.

The transitions of power consumption in different ONU states are shown in Figure 4. In the full active work state, ONUs keep in full consumption (*P*_active_) during the whole cycle, while ONUs stay in low power consuming (*P*_sleep_) sleep state for *K* cycles (ignore *T*_gates_ before falling asleep) in cyclic sleep state. In the intracycle sleep state and listening state, ONUs have low power consumption during intracycle sleep, but when the idle durations are too short to perform intracycle sleep and when ONUs wait for the GATE frames and perform data transmission (*T*_traffic_), ONUs keep in full consumption. The trapezium filled by dots depicts the power transition bank. The transition bank appears whenever switching between full consumption and sleep happens. Power consumption of this bank is set to *P*_active_ in the following sections, so the worst case is chosen.

### 2.3. Sleep Control and DBA Algorithm in the OLT

Running in the OLT periodically, the sleep control and DBA algorithm is used to decide next states of ONUs and allocate upstream and downstream bandwidth among ONUs. The algorithm is executed after the OLT has collected all REPORTs and occupancy of local downstream caches in each cycle. According to the results of the algorithm, the OLT generates the GATE frames and sends them together with downstream data to ONUs.  Pseudocode 1 is the pseudocode of the algorithm. The algorithm can be divided into four parts: cyclic sleep control, bandwidth allocation, start time arrangement, and intracycle sleep control.

**Pseudocode 1:**
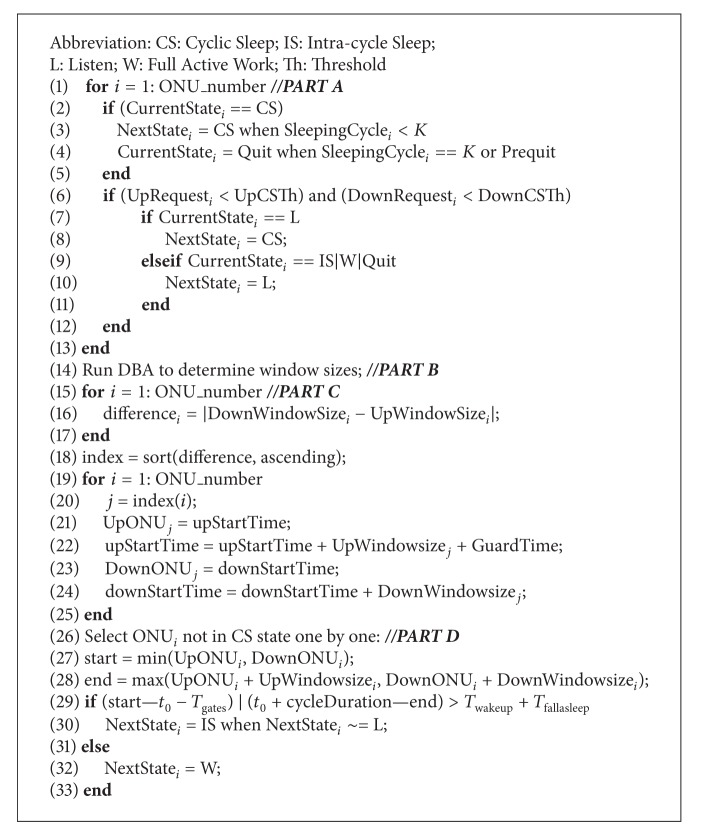
Pseudocode of the sleep control and DBA algorithm.

#### 2.3.1. Cyclic Sleep Control

The OLT controls ONUs to enter, keep in, or quit cyclic sleep states. For ONUs in cyclic sleep state, when ONUs do not finish sleeping of *K* cycles, they keep in sleep. When ONUs have kept in cyclic sleep for *K* cycles or prequitted cyclic sleep, the OLT can remove them from cyclic sleep state. According to cyclic sleep thresholds, cyclic sleep state is assigned to ONUs under light load for ONUs in listening state. ONUs under light traffic load are forced to listening state when the current states of ONUs are not listening state and cyclic sleep state.

#### 2.3.2. Bandwidth Allocation

The OLT allocates bandwidth among all ONUs by determining the sizes of upstream and downstream transmission windows. The window sizes are determined by collected upstream requests, local downstream cache occupancy, available bandwidth resource, and service level agreement (SLA) of subscribers. The DBA algorithm designed for carrying variable-rate bursty traffic which requires average rate guarantee is used for the bandwidth allocation [28]. Note that OLT should reserve bandwidth for prequitting ONUs.

#### 2.3.3. Start Time Arrangement

The OLT arranges the start time for downstream and upstream windows of ONUs. To obtain longer intracycle sleep, the OLT tries its best to align the downstream and upstream window of the same ONU. The OLT calculates the differences of downstream and upstream window sizes and sorts the differences from small to large. Then, the OLT arranges the start time for transmission windows based on the sequence of the differences. The ONU of the smallest difference can perform data transmission first. As shown in Figure 2, its upstream window and downstream window start time are

(1) $$\begin{array}{c} \text{upStartTime}=\text{downStartTime}+T_{2},\\ \text{downStartTime}=t_{0}+T_{1}+T_{\text{gates}}, \end{array}$$

where *t*_0_ is the start of each scheduling cycle. *T*_1_ is the propagation delay between the OLT and an ONU. *T*_2_ is the sum of response and requisite delay. Response delay is for ONUs to process the GATE frames and requisite delay compensates variation of propagation delay. Upstream and downstream windows of other ONUs are arranged one by one. Guard time is inserted in the middle of any two upstream windows.

#### 2.3.4. Intracycle Sleep Control

The OLT checks the length of idle durations to control the entrance of intracycle sleep. Since the time information of transmission windows is required, this step is performed after Sections 2.3.2 and 2.3.3. When idle durations are long enough for an ONU to fall asleep and wake up in time, intracycle sleep is assigned to the ONU; otherwise the ONU keeps full active.

## 3. Mathematical Model and Numerical Results

In this section, the power saving and delay performance of the hybrid sleep are theoretically analyzed via Markov chains. In this section we only consider the upstream traffic and ONU does not prequit sleep. The impacts of downstream traffic and prequitting method are taken into account in Section 4. It is assumed that the packet arrival is independent and is not affected by previous arrived packets, so the Poisson process is used to simulate the traffic arrival. Arriving packets are stored in FIFO (first in first out) queues. A simplified on-demand DBA algorithm controls the departure of queued packets.

### 3.1. States and Transition Probability Matrix

In the model, **Q** = {**W**, **I** **S**, **L**, **C** **S**} is the set of ONU states, where **W** = {*w*(*n*), *M* < *n* ≤ Size_FIFO_}, **I** **S** = {is(*n*), *N* ≤ *n* ≤ *M*}, **L** = {*l*(*n*), 0 ≤ *n* < *N*}, and **C** **S** = {cs(*n*), 0 ≤ *n* < *N*} are the subsets of full active work states, intracycle sleep states, listening states, and cyclic sleep states, respectively. *n* is the number of packets stored in the FIFO queue of an ONU at the beginning of each state. *M* and *N* are the thresholds of intracycle sleep and cyclic sleep. Size_FIFO_ is the maximum stored packet number in FIFO queue of an ONU.

**Figure 5:**
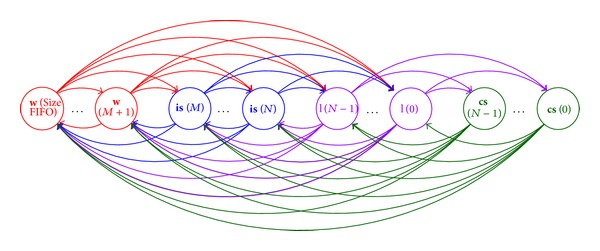
ONU state transitions in the mathematical model.

Let *X*_*i*_ be the state of one arbitrary ONU after the reception of the *i*th GATE frame; then *X*_*i*_ belongs to the state set **Q** and {*X*_*i*_, *i* = 1,2,…} is a stochastic process. Therefore, in the hybrid sleep mechanism, the state transitions from state *X*_*i*_ to state *X*_*i*+1_ can be expressed as in Figure 5. In the figure, the beginning of one arrow is connected to *X*_*i*_ and the end of an arrow points to *X*_*i*+1_. After any state *X*_*i*_, an ONU switches into full active work states when the number of queued upstream packets of *X*_*i*+1_ satisfies *n* > *M*. For *N* ≤ *n* ≤ *M*, an ONU enters intracycle sleep. When 0 ≤ *n* < *N*, an ONU enters listening states from the other three states, while from listening states an ONU transfers into cyclic sleep states. Since no packets are sent during cyclic sleep states, at the end of cyclic sleep states ONUs go to other states of more queued packets. It can be seen that for any *i* > 0 and known *X*_*i*_ = *q*(*n*), *q*(*n*) ∈ **Q** the probability of *X*_*i*+1_ = *q*(*m*), *q*(*m*) ∈ **Q** is only related to *X*_*i*_ = *q*(*n*) and is not affected by the states of the ONU before the *i*th state *X*_*i*_. Therefore, {*X*_*i*_, *i* = 1,2,…} is a Markov chain.

Let *λ* be the average arrival packet number from users per cycle. Setting the initial time to 0, for Poisson traffic, the probability of *j* packets arriving at queue during *β* cycles is

(2) $$\begin{array}{c} \text{p}{\text{r}}^{a}\left(j,\lambda \beta \right)=\{\begin{array}{cc} \frac{{\left(\lambda \beta \right)}^{j}\exp\left(-\lambda \beta \right)}{j!}, & j\geq 0\\ 0, & j<0. \end{array} \end{array}$$

The simplified on-demand DBA controls the departure of packets and follows the below allocation principles. When the bandwidth request is smaller than fixed assured/maximum bandwidth, bandwidth allocated to an ONU is equal to its request; otherwise, assured bandwidth is assigned to the ONU. No bandwidth is allocated to an ONU in cyclic sleep state. Ignore the propagation delay between the OLT and an ONU. Suppose that at the beginning of state *X*_*i*_ = *q*(*n*) the OLT gets current queued packet number *n* and uses it as bandwidth request. *μ* denotes the assured upstream departure packet number per cycle of an ONU. State *X*_*i*_ lasts *β* scheduling cycles. The probability of *j* packets leaving queue during *X*_*i*_ is

(3) $$\begin{array}{c} \text{p}{\text{r}}^{d}\left(j,\mu \beta ,n\right)=\{\begin{array}{cc} 1, & j=n,\;\;n<\mu \beta \\ 1, & j=\mu \beta ,\;\;n\geq \mu \beta \\ 0, & \text{otherwise}. \end{array} \end{array}$$

Therefore, the one-step state transition probability from state *X*_*i*_ = *q*(*n*), *q*(*n*) ∈ **Q** to state *X*_*i*+1_ = *q*(*m*), *q*(*m*) ∈ **Q** is

(4) $$\begin{array}{c} p_{q(n)q(m)}=\{\begin{array}{cc} \gamma_{1}, & \text{if}\;q\left(n\right)\in W\cup IS,\;\;q\left(m\right)\in Q-CS\\ \gamma_{2}, & \text{if}\;q\left(n\right)\in CS,\;\;q\left(m\right)\in Q-CS\\ \gamma_{1}, & \text{if}\;q\left(n\right)\in L,\;\;q\left(m\right)\in Q-L\\ 0, & \text{otherwise}, \end{array} \end{array}$$

where the first line is the transition probability from full active work states and intracycle sleep states to all states except for cyclic sleep states. The two states cannot transfer to cyclic sleep states as shown in Figure 5. The second line is the transition probability from cyclic sleep states to other states. The transition probability from listening states to other states is shown in the third line. *γ*_1_ = ∑_*j*=0_^*∞*^pr^*d*^(*j*, *μ* × 1, *n*)pr^*a*^(*j* + *m* − *n*, *λ* × 1) and it means that when the total number of queued packets changes from *n* to *m*, there are (*j* + *m* − *n*) packets arriving for any given *j* ≥ 0 removed packets during state *X*_*i*_. Except for cyclic sleep states the duration of other states is 1 cycle, so *β* = 1 for *γ*_1_. Because no packets leave upstream queue during cyclic sleep states, *γ*_2_ = pr^*a*^(*m* − *n*, *λ* × *K*) and it is expressed that (*m* − *n*) new packets are stored in queue during the *K* sleeping cycles.

Based on (4), the one-step transition probability matrix of Markov chain {*X*_*i*_, *i* = 1,2,…} with state space **Q** can be defined as

(5) $$\begin{array}{c} P=\left\{p_{q(n)q(m)},q\left(n\right)\in Q,q\left(m\right)\in Q\right\}. \end{array}$$

Because there are Size_FIFO_ + *N* + 2 elements in **Q**, **P** is a (Size_FIFO_ + *N* + 2) × (Size_FIFO_ + *N* + 2) matrix.

### 3.2. Steady-State Probabilities

With transition probability matrix, the steady-state probabilities of all states are derived in this section and used to calculate average power consumption and queuing delay in Section 3.3. ***π*** = {*π*_*q*(*n*)_, *q*(*n*) ∈ **Q**} is defined as the steady-state probability array of all Size_FIFO_ + *N* + 2 states in **Q** when the network has run for sufficient long time and stays in steady state. For the steady-state probability array of the Markov chain, the following equation should be met:

(6) $$\begin{array}{c} \pi P=\pi . \end{array}$$

The sum of all steady-state probabilities should be 1, so (7) is obtained:

(7) $$\begin{array}{c} \overset{\text{all}}{\underset{q\left(n\right)\in Q}{\sum }}\pi_{q(n)}=1. \end{array}$$

The steady-state probabilities of all states can be achieved by solving (6) and (7). To solve (6) and (7), (6) and (7) are expressed in the following matrix form:

(8) $$\begin{array}{c} \pi \left(P-I\right)=0,\\ \pi 1^{T}=1, \end{array}$$

where **I** is a (Size_FIFO_ + *N* + 2) × (Size_FIFO_ + *N* + 2) identity matrix whose diagonal elements are 1. 0 and 1 are all 0 and all 1, 1 × (Size_FIFO_ + *N* + 2) matrix, respectively. The superscript *T* means the transpose of a matrix. Next, (8) can be merged into

(9) $$\begin{array}{c} \pi A=Y, \end{array}$$

where $A=\left[\begin{array}{cc} \left(P-I\right) & 1^{T} \end{array}\right]$ and $Y=\left[\begin{array}{cc} 0 & 1 \end{array}\right]$. Other elements of **Y** are 0, except that the last element is 1. **A** is a (Size_FIFO_ + *N* + 2) × (Size_FIFO_ + *N* + 3) matrix. Therefore, the steady-state probabilities can be obtained by

(10) $$\begin{array}{c} \pi =\frac{Y}{A}. \end{array}$$

The results of matrix division can be achieved with the help of MATLAB and only second-level running time is needed.

### 3.3. Power Consumption and Delay

Let ${\bar{E}}_{\text{ONU}}$ and ${\bar{P}}_{\text{ONU}}$ be the average energy and power consumption of ONUs. Using the steady-state probabilities,

(11) E¯ONU=Pactive∑q(n)∈Wallπq(n)+∑q(n)∈ISallπq(n)P{q(n)} +∑q(n)∈Lallπq(n)P{q(n)}+Psleep∑q(n)∈CSallπq(n)×K,P¯ONU=E¯ONU{∑q(n)∈W∪IS∪Lallπq(n)+∑q(n)∈CSallπq(n)×K},

where *P*{*q*(*n*)} are the power consumption of intracycle sleep and listening states:

(12) $$\begin{array}{c} P\left\{q\left(n\right)\right\}=\frac{[P_{\text{active}}T_{\text{active}}+P_{\text{sleep}}\left(T_{\mathrm{cycle}}-T_{\text{active}}\right)]}{T_{\mathrm{cycle}}}, \end{array}$$

where *q*(*n*) ∈ **I** **S** ∪ **L** and the total full consuming duration is *T*_active_ = *T*_gates_ + *T*_traffic_ + 2 × (*T*_wakeup_ + *T*_fallasleep_). As shown in Figure 4, *T*_gates_, *T*_traffic_, *T*_wakeup_, and *T*_fallasleep_ are the durations of receiving the GATE frames, data transmission, waking up periods, and falling asleep periods, respectively. Furthermore, power saving rate is defined as

(13) $$\begin{array}{c} \eta =\frac{\left(P_{\text{active}}-{\bar{P}}_{\text{ONU}}\right)}{P_{\text{active}}}. \end{array}$$

Next, to calculate the average upstream queuing delay ${\bar{D}}_{\text{up}}$, it is supposed that *n* packets are in queue of an ONU in the start of state *X*_*i*_ = *q*(*n*), *j* packets arrive at queue during *β* cycles of *X*_*i*_, and *μ* is the assured departure rate. Each packet is sent after all prior arriving packets are sent. All the *j* new arrival packets experience 4 durations before leaving queue.

First, according to the principle of the simplified DBA, the *j* new arrival packets during state *X*_*i*_ will not be sent until state *X*_*i*+1_. Because the *j* packets arrive at queue at random, they have to wait for *β*/2 scheduling cycles in average before the start of state *X*_*i*+1_.

Second, let *n*_send_ be the number of transmitted packets during *X*_*i*_ = *q*(*n*); then *n*_send_ = min⁡(*n*, *μ*). Specially, for *X*_*i*_ = *q*(*n*) ∈ **C** **S**, *n*_send_ = 0. Therefore, the *h*th (0 < *h* ≤ *j*) new arrival packet will be sent in the *C*_send_th = ceil[(*n* − *n*_send_ + *h*)/*μ*] scheduling cycle after state *X*_*i*_.

Third, within the *C*_send_th scheduling cycle, the *h*th packet needs to wait for the upstream window allocated to designated ONU before the departure of queue. Under different traffic loads (*ρ*), the average time of waiting for the upstream window varies. For example, under light load the windows of all ONUs locate in the front of each scheduling cycle and the average waiting time is short. *T*_cycle⁡_ denotes the duration of one scheduling cycle. The sequence of windows of different ONUs is random, so the average waiting time for upstream window within the *C*_send_th scheduling cycle is *ρT*_cycle⁡_/2.

Finally, in the upstream window, the *h*th packet waits for the departure of packets that share the same window and arrive at the queue of the same ONU earlier. Packets leave queue at line rate of 10G-EPON, so the average value of this delay (several microseconds) is negligible comparing to the ms-level total queuing delay.

Therefore, for the *j* new arrival packets in state *X*_*i*_ = *q*(*n*), the average queuing delay is

(14) D¯(j ∣ n,β) =βTcycle⁡2+∑h=1j[(Csend−1)×Tcycle⁡+ρTcycle⁡/2]j, j≠0,

and the average upstream queuing delay of all states is equal to

(15) D¯up =∑q(n)∈W∪IS∪Lallπq(n)∑j=1SizeFIFOpra(j,λ)D¯(j ∣ n,1)  +∑q(n)∈CSallπq(n)∑j=1SizeFIFOpra(j,λK)D¯(j ∣ n,K).

### 3.4. Numerical Results

The numerical results of steady-state probability, power saving rate, and upstream queuing delay are calculated under different traffic loads in this section. The impacts of critical parameters including intracycle sleep threshold and cyclic sleep threshold on the performance of the proposed mechanism are studied. Moreover, comparisons between the proposed mechanism and existing cyclic sleep mechanism and pure intracycle sleep mechanism are carried on.

Table 2 shows the parameters used in both this section and Section 4. Besides those parameters, it is assumed that the 10G-EPON system consists of an OLT and 16 ONUs. As Ethernet packet size is in the scope of [64,1518] bytes, the average packet size is set to 800 bytes. Each ONU has the same average arrival packet number per cycle *λ*; then the traffic load of the 10G-EPON network is *ρ* = *λ* × 800 bytes × 16/2 ms × 10 Gbps. The assured bandwidth of an ONU is set to the value when the total upstream bandwidth is equally distributed to all ONUs, so *μ* = 10 Gbps × 2 ms/16 × 800 bytes ≈ 200 packets/cycle is the assured departure packet number per cycle. When the size of queue is 10 Mbytes, Size_FIFO_ = 10*M*/800 = 12500 packets. This value is big enough to cover most packet arrival and departure cases and does not make the scale of Markov chains too large to be solved. When the intracycle sleep threshold

(16) $$\begin{array}{c} M=\frac{\left[T_{\mathrm{cycle}}-T_{\text{gates}}-2\times \left(T_{\text{wakeup}}+T_{\text{fallasleep}}\right)\right]\times 10\;\text{Gbps}}{800\;\text{bytes}} \end{array}$$

is not exceeded, an ONU can still enter intracycle sleep state. *T*_traffic_ is calculated with the number of transmitted packets in each state. *T*_fallasleep_ is set to the value of *T*_wakeup_.

**Figure 6:**
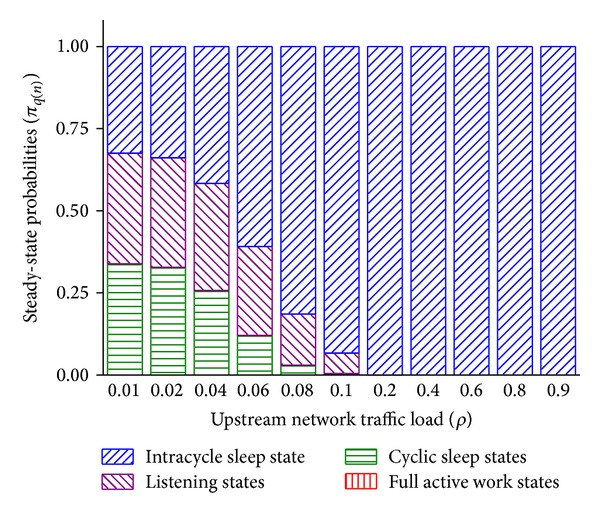
Steady-state probabilities of different traffic loads.

In Figure 6, to verify the correctness of the model, the steady-state probabilities of the four sets of states are shown. Cyclic sleep threshold *N* is fixed to 12 packets and it is equivalent to 12 × 800 bytes/2 ms ≈ 40 Mbps. *T*_wakeup_ is fixed to 2 *μ*s. The sum of all steady-probabilities is 1 under any load. (a) The sum of steady-state probabilities of intracycle sleep states increases together with the traffic load, has a notable boost around 0.06 load, and reaches 1 after 0.2 load. The steady-state probabilities of listening and cyclic sleep states have reverse trends. (b) For all traffic loads, the probability sum of full active work states is near 0 as expected. The reasons are that (a) higher traffic load makes the entrance of cyclic sleep harder, so the ONU tends to perform intracycle sleep. Under 0.06 load, the average arrival rate of an ONU is 10 Gbps × 0.06/16 = 37.5 Mbps and the 40 Mbps cyclic sleep threshold becomes easier to be exceeded. After 0.2 load, because the 125 Mbps average arrival rate is far beyond the cyclic sleep threshold and the Poisson traffic is steady relatively, the ONU hardly enters listening and cyclic sleep states. (b) *M* = [2 ms − 10 *μ*s − 2 × (2 + 2) *μ*s] × 10 Gbps/800 bytes ≈ 3100 packets is the threshold for entering intracycle sleep. *λ* = 180 packets/cycle under 0.9 load. Therefore, for example, even under 0.9 load, according to (4) the probability of transition from *q*(0) ∈ **C** **S** to *q*(*M* + 1) ∈ **W** is pr^*a*^(3101,180 × 10) ≈ 0. It is hard to exceed intracycle sleep threshold and enter full active work states.

**Figure 7:**
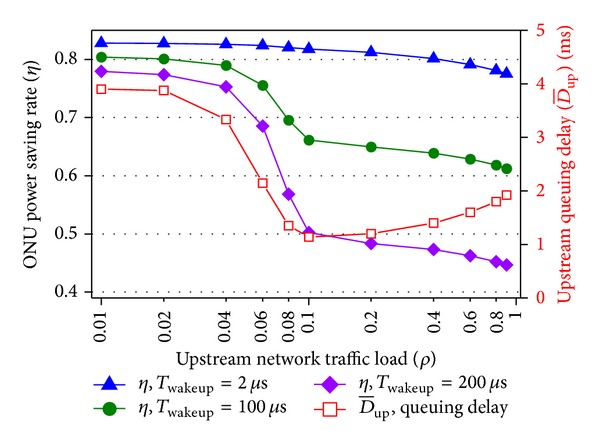
ONU power saving rate and delay versus traffic load and wake-up time (mathematical model).

In Figure 7, the power saving rate and delay under different traffic loads and the impacts of intracycle sleep threshold on performances are shown. By setting *T*_wakeup_ to 2, 100, and 200 *μ*s, the intracycle sleep threshold is changed together. Other parameters keep unchanged. First, when *T*_wakeup_ is equal to 2 *μ*s, at least 77.52% consumption is preserved even under heavy traffic load. The hybrid sleep mechanism based on fast CDR circuit is effective even under heavy traffic load. Then, the power saving rate decreases with the increase of traffic load. This is because more time is used for data transmission. Third, *T*_wakeup_ has less impacts on the power saving rate for lighter load. With higher *T*_wakeup_ more energy is wasted in the wake-up and fall-asleep transition banks to perform intracycle sleep, but when traffic load is below 0.06, the ONU tends to perform cyclic sleep instead of intracycle sleep and the wasted energy is saved. Finally, (a) the delays with different *T*_wakeup_ are the same. (b) With the raising of traffic load, the delay goes down first and then up. The reasons are that (a) intracycle sleep utilizes idle durations between transmissions and does not postpone packet departure. *T*_wakeup_ only has impact on intracycle sleep, so the delays are the same. (b) Under light load, cyclic sleep is active and higher delay is achieved to wait for the end of *K* sleeping cycles. The adoption of cyclic sleep sacrifices the delay performance. Under higher traffic, cyclic sleep is seldom performed and the delay goes down. As the traffic load goes up further, only intracycle sleep is used and more queued packets incur higher delay.

**Figure 8:**
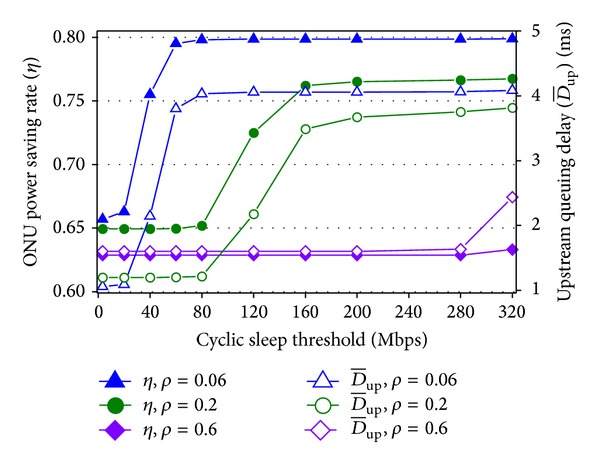
ONU power saving rate and delay versus traffic load and cyclic sleep threshold.

In Figure 8, the impacts of cyclic sleep threshold on the power saving rate and delay are studied. Cyclic sleep threshold *N* is varied from 1 to 100 packets and is equivalent to 3.2–320 Mbps. The traffic load is set to 0.06, 0.2, and 0.6, respectively, so the corresponding average ONU arrival rate is 37.5, 125, and 375 Mbps. *T*_wakeup_ is fixed to 100 *μ*s. First, under 0.06 load the power saving rate increases with *N*, has a sudden rise around 40 Mbps threshold, and has the roof. The reasons are that when the threshold is lower than the 37.5 Mbps arrival rate, the ONU seldom enters cyclic sleep and the low power saving rate is determined by the ability of intracycle sleep with 100 *μ*s *T*_wakeup_. When the threshold is around 40 Mbps, cyclic sleep is adopted easier. The power saving rate is improved via cyclic sleep. The roof of power saving rate is caused by the existence of data transmission and overhead durations. The changes of delay have similar trends and causes. Second, under 0.2 load, the sudden rises appear when the cyclic sleep threshold is around the 125 Mbps arrival rate. The power saving rate of 0.2 load is less than 0.06 load, because under heavier load there are less opportunities to enter cyclic sleep and more data to be transmitted. Third, under 0.6 load the 375 Mbps arrival rate is too high for ONUs to enter cyclic sleep in the selected scope of cyclic sleep threshold, so the power saving rate is determined by intracycle sleep and is low for most values of the threshold. With small thresholds, the delay under 0.6 load is higher, because there are more traffic data and cyclic sleep is not performed. However, with large thresholds it is still hard for ONUs to enter cyclic sleep under 0.6 load, so the delay of 0.6 load is lower than the other two loads.

**Figure 9:**
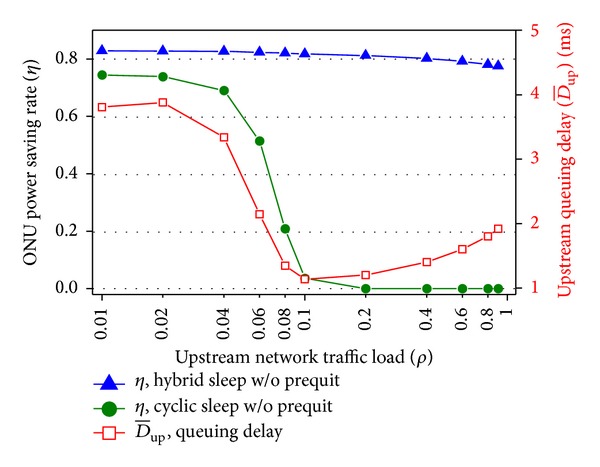
Performance comparison between proposed hybrid sleep and existing cyclic sleep.

Figure 9 illustrates the comparison between the proposed hybrid sleep and existing cyclic sleep [18]. The performance of existing cyclic sleep is obtained by removing intracycle sleep states from the model. *T*_wakeup_ is 2 *μ*s. The cyclic sleep threshold is set to 40 Mbps. The power of sleep mode in existing cyclic sleep mechanism is 0.7 W [11]. The power saving rate of existing cyclic sleep keeps decreasing and reaches 0 under heavy traffic. However, for hybrid sleep, the power saving rate is always higher than the other case. Especially, under heavy load the existing cyclic sleep does not take effect, while the power saving rate of hybrid sleep still holds high value. Actually, under 0.01 and 0.2 load, the minimum and maximum differences of power saving rate between the proposed hybrid sleep and existing cyclic sleep are 0.08 and 0.81. Thus, in average 44.5% power consumption is further reduced by adopting sleep within a scheduling cycle. Also, the average queuing delays of the two mechanisms are the same. Compared to existing cyclic sleep the proposed mechanism can further reduce energy consumption with little QoS degradation even under heavy traffic. The above conclusions result in the fact that the hybrid sleep utilizes idle durations among transmissions. This way creates more chances for sleep even under heavy traffic and does not postpone data transmission.

**Figure 10:**
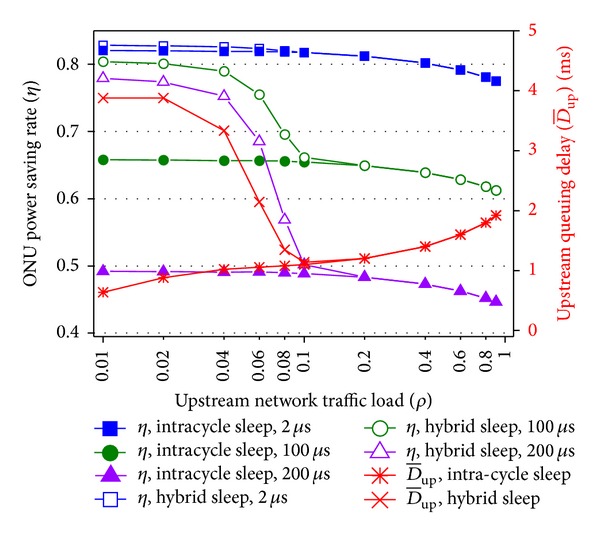
Performance comparison between proposed hybrid sleep and pure intracycle sleep.

In Figure 10, comparison between the proposed hybrid sleep and pure intracycle sleep is performed. Listening and cyclic sleep states are deleted to calculate the performance of pure intracycle sleep. *T*_wakeup_ is set to 2, 100, and 200 *μ*s. The cyclic sleep threshold is still 40 Mbps. First, under light load the power saving rates of hybrid sleep are higher than the pure intracycle sleep and with the increasing of *T*_wakeup_ the difference is enlarged. The hybrid sleep improves the power saving rate under light load. Under heavy load, the two mechanisms have equal power saving rate. This is because under light traffic the pure intracycle sleep can only perform short intracycle sleep and wastes energy in overhead durations, but the hybrid sleep can take long cyclic sleep. With the increasing of *T*_wakeup_, overhead durations become longer and waste more energy, so the effect of taking long cyclic sleep becomes more notable. Under heavy traffic, the hybrid sleep cannot perform cyclic sleep to reduce overhead durations any more. Second, under light load the upstream delay of hybrid sleep is higher than intracycle sleep. Hybrid sleep has better power saving performance in the price of higher delay under light traffic. However, as long as the delay meets QoS requirement, this price can be taken.

## 4. Simulation System and Results

To validate the accuracy of the mathematical model, a simulation system of 10G-EPON is established by using MATLAB. Although most of analyses in Section 3.4 are performed again by the simulation system, for concision only the impacts of intracycle sleep threshold on performances are shown by simulations in this section. In addition, the performance of the hybrid sleep with prequitting method, which is not considered in the mathematical model, is studied. The sleep control and DBA algorithm expressed in Section 2.3 is used in the simulation system.

Besides parameters listed in Table 2, other simulation parameters are set as follows. 7500 cycles are run for each simulation. The arrival traffic is of self-similar characteristics with 0.8 Hurst parameter. For each ONU, there are one low priority and the other high priority traffic link for both upstream and downstream. High priority traffic takes up to 5% of the total traffic. All ONUs have equal upstream and downstream average arrival rate. The network traffic load is defined as the ratio of total arrival rate and the capacity of both transmission directions. One 10 Mbytes FIFO queue is used to store packets for each traffic link. No packet is dropped in all simulations. The power of sleep mode for existing cyclic sleep mechanism is 0.7 W [11]. The cyclic sleep threshold is set to 40 Mbps. *T*_wakeup_ is 2 *μ*s.

**Figure 11:**
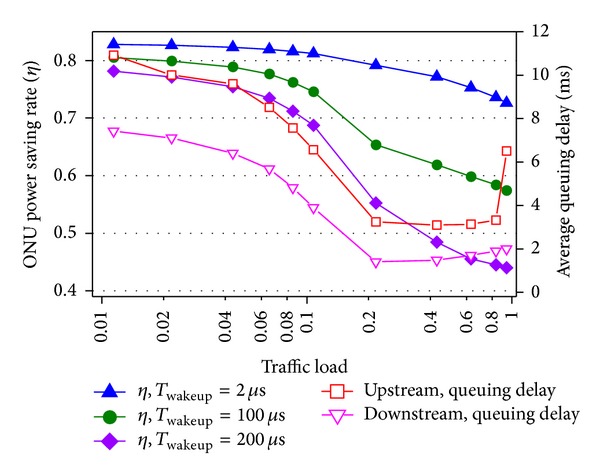
ONU power saving rate and delay versus traffic load and wake-up time (simulation system).

In Figure 11, the impacts of intracycle sleep threshold on the performances are analyzed again based on the simulation system. First, the power saving rate and upstream delay curves here have similar trends as results in Figure 7 obtained by using the mathematical model. It verifies the accuracy of the mathematical model. Second, compared to upstream delay curve in Figure 7, the upstream delay here has a great increase at 0.9 traffic load. This difference lies in the fact that self-similar traffic has stronger burstiness than Poisson traffic. Third, the downstream delay is lower than the upstream delay. This is because all downstream traffic arrives at the OLT from one 10 Gbps SNI and leaves the OLT by one 10 Gbps PON interface. There is no downstream bandwidth contention among ONUs.

**Figure 12:**
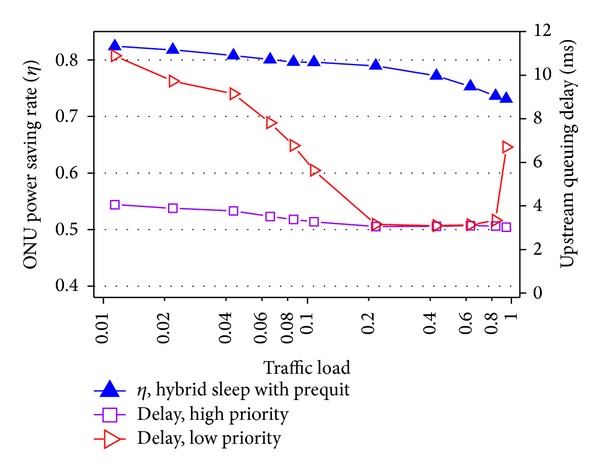
Performance of hybrid sleep with prequit method.

Figure 12 illustrates the performance of the proposed hybrid sleep with prequitting method [19]. First, the delay of upstream high priority traffic is hardly affected by the changing of traffic load, while the delay of low priority traffic is high under light load and jumps to high value again under 0.9 traffic load. Under light traffic, the prequitting method distinguishes the delay of high and low priority. Under 0.9 traffic load, the packet schedule strategy in ONUs of sending high priority packets first when the bandwidth resource is not enough leads to the above difference. Second, the power saving rate is of little decline compared to Figure 11 without prequitting method. This is because of the entrance of intracycle sleep instead of entering full active state after prequitting cyclic sleep. Therefore, in the proposed mechanism, the delay guarantee of high priority traffic can be obtained with little sacrifice of power dissipation.

## 5. Conclusion

Based on ONUs with fast clock recovery ability, the proposed hybrid mechanism can reduce energy consumption even under heavy traffic load with QoS guarantee and traffic differentiation. A mathematical model is established to evaluate power saving rate and queuing delay of ONUs in the proposed mechanism and perform performance comparison with existing approaches. The numerical results of the model demonstrate that at least 77.52% power consumption can be reduced with little QoS degradation even under heavy traffic load. Compared to existing cyclic sleep mechanism, in average, 44.5% power consumption is further reduced. Compared to pure intracycle sleep, the power saving rate is improved under light traffic by the mixture of cyclic and intracycle sleep with increase of delay especially for ONUs of long wake-up time. Simulation results verify the accuracy of the mathematical analysis and amplify that the delay of high priority traffic is guaranteed with little sacrifice of power dissipation.

## Figures and Tables

**Table 1:** Overhead time during wake-up process.

Name of overhead time	Duration
Laser on time	512 ns
Receiver setting time	800 ns
Clock recovery time	500 ns
Frame synchronization time	0
Total overhead time	<2 *μ*s

**Table 2:** Common parameters used in both mathematical model and simulation system.

Name of parameters	Value
Line rate	10 Gbps
Traffic load (ρ)	0.01 to 0.9
Duration of scheduling cycle (*T*_cycle⁡_)	2 ms
Time for receiving GATE frames (*T*_gates_)	10 *μ*s
Number of sleeping cycles (*K*)	10 cycles
Power of full consumption (*P*_active_)	6.35 W [11]
Power of sleep mode for hybrid sleep mechanism (*P*_sleep_)	1.08 W [12]
